# MYLK*FLNB and DOCK1*LAMA2 gene–gene interactions associated with rheumatoid arthritis in the focal adhesion pathway

**DOI:** 10.3389/fgene.2024.1375036

**Published:** 2024-05-13

**Authors:** Maëva Veyssiere, Maria del Pilar Rodriguez Ordonez, Smahane Chalabi, Laetitia Michou, François Cornelis, Anne Boland, Robert Olaso, Jean-François Deleuze, Elisabeth Petit-Teixeira, Valérie Chaudru

**Affiliations:** ^1^ Institut National de la Santé et de la Recherche Médicale, Université de Paris, Paris, France; ^2^ GenHotel—Univ Evry, University of Paris Saclay, Evry, France; ^3^ Division of Rheumatology, Department of Medicine, CHU de Québec-Université Laval, Québec City, QC, Canada; ^4^ Génétiqe-Oncogénétique Adulte-Prévention, Institut National de la Santé et de la Recherche Médicale, Clermont-Auvergne University and CHU, Clermont-Ferrand, France; ^5^ Commissariat à l'Energie Atomique, Centre National de Recherche en Génomique Humaine (CNRGH), Université Paris-Saclay, Evry, France

**Keywords:** rare variants, pathway enrichment analysis, gene–gene interaction, rheumatoid arthritis, familial sample

## Abstract

Rheumatoid arthritis (RA) is a chronic, systemic autoimmune disease caused by a combination of genetic and environmental factors. Rare variants with low predicted effects in genes participating in the same biological function might be involved in developing complex diseases such as RA. From whole-exome sequencing (WES) data, we identified genes containing rare non-neutral variants with complete penetrance and no phenocopy in at least one of nine French multiplex families. Further enrichment analysis highlighted focal adhesion as the most significant pathway. We then tested if interactions between the genes participating in this function would increase or decrease the risk of developing RA disease. The model-based multifactor dimensionality reduction (MB-MDR) approach was used to detect epistasis in a discovery sample (19 RA cases and 11 healthy individuals from 9 families and 98 unrelated CEU controls from the International Genome Sample Resource). We identified 9 significant interactions involving 11 genes (*MYLK*, *FLNB*, *DOCK1*, *LAMA2*, *RELN*, *PIP5K1C*, *TNC*, *PRKCA*, *VEGFB*, *ITGB5*, and *FLT1*). One interaction (MYLK*FLNB) increasing RA risk and one interaction decreasing *RA risk* (DOCK1*LAMA2) were confirmed in a replication sample (200 unrelated RA cases and 91 GBR unrelated controls). Functional and genomic data in RA samples or relevant cell types argue the key role of these genes in RA.

## Introduction

Rheumatoid arthritis (RA) is a chronic, systemic autoimmune disease affecting approximately 0.5 per cent of the population worldwide (Almutairi et al., 2021). Although the exact etiology of the disease remains unclear, a combination of genetic and environmental factors seems crucial for its development. Currently, over a hundred genetic loci have been associated with increased RA risk, including the *HLA-DRB1* gene and its shared epitope (SE) alleles (McAllister et al., 2011; van Drongelen and Holoshitz, 2017). These discoveries have been made possible, thanks to the evolution of genome analyses, from genome-wide association studies (GWAS) to next-generation sequencing (NGS) technologies, accompanied by a shift in the tested hypothesis from frequent variants alone (McAllister et al., 2011; Ha et al., 2021) to a combination of all frequency variants including rare variants (Diogo et al., 2013; Bang et al., 2014; Mitsunaga et al., 2015; Veyssiere et al., 2019a). However, most of these studies identified association signals due to variants with high deleterious effects. Yet rare variants’ accumulation with less important predicted effects in genes participating in the same biological function might be involved in the development of complex diseases such as RA.

These functions might be identified using two types of approaches: (1) a candidate approach for which the user targets a specific biological pathway identified from an *a priori* knowledge (such as pathophysiological mechanisms involved in the studied disease) and (2) a whole-genome approach where all the pathways present in a database are analyzed without *a priori*. Thanks to the small number of pathways to evaluate with the first approach, it is possible to manually curate each gene to ensure the annotation quality. Moreover, the time and resources needed to analyze such a low number of pathways are reduced compared to those required by the whole-genome approach. However, the second approach allows detecting new associations between a biological function and disease or between diseases.

Using a candidate approach, Mitsunaga et al. highlighted an aggregation of rare variants, with moderate-to-high impact, in the Japanese population affected by RA, in genes participating in the mitochondrial respiratory chain (Mitsunaga et al., 2015). A burden test combining all variants showed the association with severe erosion (OR = 2.16 [1. 43–3.28] and *p*-value = 1.56*10^–4^). More recently, an analysis of 58 cases and 66 controls from the Chinese population highlighted the enrichment of rare and frequent variants associated with RA in several biological pathways, including extracellular matrix receptor interaction, protein digestion and absorption, and focal adhesion (Li et al., 2017).

In this study, we used a whole-exome approach to identify biological functions enriched in rare variants co-segregating with RA in multiplex families. In addition, we hypothesized that interactions between the genes participating in these functions would increase the risk of developing the disease. A discovery and a replication step allowed validating this hypothesis.

## Material and methods

### Sample collection

Blood samples were obtained from 30 individuals belonging to nine French families with multiple cases of autoimmune diseases (Supplementary Figure S1). Among the 30 individuals, 19 were RA cases (26.3% had Sjögren’s syndrome in addition to RA; the others were RA cases without any other autoimmune disease), and 11 were unaffected with RA (27.3% of them had another autoimmune disease—details in Supplementary Figure S1). Data of additional healthy controls were downloaded from the International Genome Sample Resource (IGSR): 98 CEU (Utah residents with Northern and Western European ancestry) and 91 GBR (British in England and Scotland) individuals sequenced on an Illumina platform (Auton et al., 2015; Sudmant et al., 2015). The mean sequencing depth for the CEU and GBR samples is 60X. For the replication step, 200 RA samples from trio families of French European ethnicity (Michou et al., 2007) were used. The study was approved by the Ethics Committees of Hôpital Bicêtre and Hôpital Saint Louis (Paris, France; CPPRB 94–40). All subjects provided written informed consent for participation in the study.

### Variant identification

Whole-exome sequencing (WES) performed on the genomic DNA samples obtained from the nine French multiplex families, and subsequent data processing was detailed in a previous study (Veyssiere et al., 2019a). The mean sequencing depth for these samples was 100X. To summarize, the exons were captured with Agilent SureSelect Human All Exon kit (V5), sequenced on an Illumina HiSeq2000 platform, and mapped to the human reference genome hg19 (Lander et al., 2001) using the Burrows–Wheeler alignment-maximal exact match (BWA-MEM) algorithm (H. Li, 2013). Duplicated reads were removed using the Picard toolkit, and variants were called with the Haplotype Caller (HC) algorithm from the Genome Analysis ToolKit (GATK) suite (McKenna et al., 2010; Van der Auwera et al., 2013). Only single-nucleotide variants (SNVs) and small indels (maximum length of 50 bp) with a total DP (read depth) ≥ 12, mapping quality (MQ) ≥ 30, quality by depth (QD) ≥ 2, FS score (phred-scaled *p*-value using Fisher’s exact test to detect strand bias) ≤ 25, and call-rate ≥95% were kept.

Variants were classified as rare when their MAF was below 1% in population of European ancestry in the 1000 Genomes Project (Phase 3, 2015 August), the Exome Aggregation Consortium project (ExAC), the Exome Sequencing project (ESP6500–6500 exomes), and the Complete Genomics project (CG69–69 individuals).

### Pathway identification

Over-representation analysis was applied to genes containing rare non-neutral (SNPEFF annotation ∈ {HIGH, MODERATE, and MODIFIER} (Cingolani et al., 2012) and/or Phred CADDscore ≥15 (Rentzsch et al., 2019)) variants with complete penetrance and no phenocopy in at least one of the nine multiplex families. It was performed using ClueGO (Bindea et al., 2009) in Cytoscape version 3.6 (Shannon et al., 2003) for two types of annotations: the Gene Ontology “biological processes” (GO BPs) and pathway databases including Reactome (Gillespie et al., 2022), KEGG pathways (Kanehisa and Goto, 2000), and WikiPathways (Kutmon et al., 2016). Gene/term annotations inferred by computational processes only (IEA for Inferred from Electronic Annotation) were removed from the analysis. *p*-values were calculated using a unilateral hypergeometric test and corrected for multiple tests with the Benjamini–Hochberg procedure. Default clustering parameters in ClueGO were kept.

### Statistical epistasis analyses

Interactions were first tested for each pair of variants fulfilling the following quality control criteria: (1) missing genotype rate below 5%, (2) no deviation from Hardy-Weinberg equilibrium in controls (*p*-value >0.01), and (3) no linkage disequilibrium between selected variants (*R* ^2^ < 0.2). The pairwise interactions were tested using model-based multifactor dimensionality reduction (MB-MDR) (Calle et al., 2010) by applying an additive genetic model while adjusting for sex (as this variable is the only one available for all samples). This method allows identifying genotypic combinations that significantly increase RA risk, grouped under the label H, and genotypic combinations that decrease RA risk, grouped under the label L. The genotypic combinations are pooled together for each category, H and L, to test their association with RA. For pairs of variants having at least one category with a false discovery rate (FDR) below 5%, the significance of the categories was evaluated with a permutation test (up to one million permutations). To assess if an interaction model was more probable than a model including the variant’s proper effect only (model “VAR1” for variant 1 and model “VAR2” for variant 2) or a model considering an independent effect of the two variants (model “VAR 1 + VAR2”), all the models were tested and compared using the Akaike information criterion (AIC). The AIC estimates the quality of statistical models and highlights the one with the best combination between the maximum likelihood and the minimum number of parameters *k*, according to the parsimony principle. The AIC was corrected (AIC_C_) to consider the small number of samples in the dataset by applying the following formula, where *n* is the sample size:

$${AIC}_{C}=AIC+\frac{2k\left(k+1\right)}{n-k-1}.$$

The models “VAR1,” “VAR2,” and “VAR1 + VAR2” were evaluated using a logistic regression adjusted on the sex with R software version 3.2.3 (R Core Team, 2022). The interaction model was retained if the differences of the AIC with each of the three other models (ΔAIC) were higher than 10 according to the Burnham and Anderson rule for interpreting the ΔAIC scores (Burnham and Anderson, 2002).

### SNP genotyping in the replication dataset

Applied Biosystems TaqMan™ SNP Genotyping Assays were used to amplify and detect polymorphisms in purified genomic DNA samples during replication. References of each assay for genotyping are indicated in Supplementary Table S3. For quality control, 10% of samples were randomly chosen to be genotyped a second time.

## Results

### Accumulation of RA-specific rare variants in a pathway regulating focal adhesion

**FIGURE 1:**
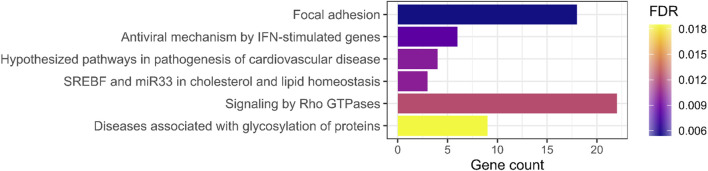
Enrichment analysis results of rare variants carried by all RA cases in at least one multiplex family. Barplot representing the number of genes carrying a rare variant (with complete penetrance and no phenocopy in nine multiplex families) by the significant biological pathway (FDR <5%). The color represents the false discovery rate (FDR) value (Benjamini–Hochberg procedure) of the enrichment analysis.

To identify biological processes that may be relevant for RA pathogenesis, we performed enrichment analysis using rare variants with complete penetrance and no phenocopy in nine multiplex families. From 1,730 rare RA-specific SNVs, 403 were non-neutral (386 SNVs and 17 indels). These variants were significantly enriched for six biological pathways (Figure 1; Supplementary Table S1). The most significant group referred to the focal adhesion pathway (FDR = 0.0054). However, it should be noted that some genes are more tolerant to non-neutral variants (e.g., with high CADD scores). Thus, the latest can accumulate even in a healthy population. To verify if the identified functions were not obtained randomly, we drew 100 sets of 403 non-neutral variants from the GnomAD healthy population and performed an enrichment analysis for each of them. Pathways associated with signaling by Rho GTPases and lipid metabolism were the only ones with significant enrichment of non-neutral variants (with, respectively, 4/100 and 1/100 significant enrichments), reinforcing our results on RA patients. The enrichment analysis performed on the GO database highlighted 22 groups of annotations, including 49 terms, significantly enriched in the list of selected genes (FDR <5%). Multiple functions linked to the regulation of T lymphocytes (differentiation, proliferation, and activation) and focal adhesion regulation were identified (Supplementary Table S2).

### Epistatic interactions associated with RA in the focal adhesion pathway

We showed an accumulation of RA rare variants in the focal adhesion pathway and hypothesized that the presence of some combination of variants in the genes participating in this pathway may increase, or reduce, the risk of developing RA. To verify this, we performed a statistical interaction analysis. We tested pairwise interactions between variants, rare and frequent, localized on the focal adhesion pathway genes using our sequenced samples and 98 CEU controls. The effect of each combination of variants on RA was estimated using logistic regression models. Considering the definition of the focal adhesion pathway in WikiPathways, composed of 202 genes, we identified 1,027 SNVs present in at least one patient or control. For the 1,000 most significant H or L categories, we applied one million permutations to assess the significance of the interaction. One hundred and forty-four pairs of variants were identified as significantly associated with RA (FDR <5%). For each of these pairs, we checked if the interaction model was better than a model where only each variant’s specific effect was considered and a model where a combined effect of the two variants was considered without interaction. We identified 52 interaction models which were better than the other models. Among those, nine interaction models had a ΔAIC >10 with all the competing models. These interactions involved 14 SNVs (Table 1).

**TABLE 1:** Significant pairwise interactions of variants associated with RA in the focal adhesion pathway using 30 individuals (19 RA and 11 controls) from multiplex families and 98 CEU controls from ISGR.

Combination		Genotype			Frequency		MB-MDR		ΔAIC with		
Gene1 × Gene2	SNV1 × SNV2	SNV1	SNV2^a^	Cat	Cases	Controls	OR [95% CI]	FDR^b^	SNV1	SNV2	SNV1 + SNV2
MYLK*FLNB	rs2605418*rs1658338	1	1	H	0.53	0.092	18.89 [5.89–60.54]	7.62E-04	−36.85	−39.78	−38.56
		0	2		0.21	0.046					
DOCK1*LAMA2	rs11017150*rs2244008	0	0	L	0.21	0.78	0.07 [0.022–0.24]	6.00E-06	−26.21	−33.89	−26.49
		0	1	H	0.47	0.094	18.3 [5.36–61.40]	3.13E-03	−30.81	−38.49	−31.09
		1	0		0.032	0.074					
RELN*MYLK	rs2229864*rs55956729	2	0	H	0.32	0.037	14.88 [4.72–46.87]	3.87E-03	−33.86	−33.73	−35.75
		0	1		0.26	0.046					
PIP5K1C*FLNB	rs542690*rs1658338	0	1	H	0.53	0.14	19.38 [5.92–65.62]	1.88E-03	−37.01	−27.58	−24.11
		0	2		0.26	0.028					
TNC*LAMA2	rs2989519*rs2244008	0	1	H	0.16	0.018	13.37 [4.3–41.35]	6.47E-03	−28.15	−32.62	−24.71
		1	1		0.21	0.0092					
		2	0		0.26	0.11					
		0	0	L	0.052	0.29	0.078 [0.023–0.26]	4.00E-06	−27.49	−31.96	−24.05
		1	0		0.16	0.47					
PRKCA*RELN	rs3803821*rs262338	0	1	H	0.42	0.073	14.23 [4.54–44.56]	4.95E-03	−32.17	−27.93	−27.34
		1	0		0.32	0.10					
VEGFB*LAMA2	rs11603042*rs2244008	0	1	H	0.32	0.37	17.05 [4.68–62.02]	1.47E-02	−21.58	−29.91	−22.98
		2	1		0.11	0.0092					
ITGB5*FLNB	rs2291079*rs1658338	0	1	H	0.58	0.17	15.24 [4.59–50.65]	8.21E-03	−29.81	−30.70	−29.72
		0	2		0.2	0.046					
FLT1*LAMA2	rs2296189*rs3778137	1	0	H	0.47	0.073	12.09 [3.77–38.76]	2.20E-02	−23.45	−26.31	−22.63

^a^ Category assigned to the genotype combination; H: increased RA risk and L: reduced RA risk.

^b^ FDR calculated by applying the Benjamini–Hochberg procedure.

### Replicated interactions between MYLK and FLNB and DOCK1 and LAMA2

We selected the five most significant gene–gene interactions (FDR <0.004—Table 1) for replication. For this purpose, we genotyped 8 participating SNVs on 200 RA patients. After adding 91 GBR controls from the IGSR, we applied the same strategy used in the discovery phase. Three interactions, MYLK*FLNB, DOCK1*LAMA2, and PIP5K1C*FLNB, were significantly associated with RA in the replication dataset (Table 2). One high-risk combination for MYLK*FLNB and two for DOCK1*LAMA2 were more probable than the concurrent models (variant proper effect and additive without interaction) in both initial and replication sets (bold in Table 2). No genotypic combination was associated with RA in the replication dataset (minimum *p*-value = 0.14) for RELN*MYLK. The significant combinations identified for PIP5K1C*FLNB differed from those identified in the multiplex families of the discovery set. As all replicated interactions harbored a ΔAIC >10, it strengthens our findings.

**TABLE 2:** Pairwise interactions in 200 RA cases and 91 GBR controls.

Combination		Genotype			Frequency		MB-MDR		ΔAIC with		
Gene1 × Gene2	SNV1 × SNV2	SNV1	SNV2	Cat^a^	Cases	Controls	OR [95% CI]	*p*-value^b^	SNV1	SNV2	SNV1 + SNV2
MYLK*FLNB	rs2605418*rs1658338	**1**	**1**	**H**	**0.38**	**0.15**	**2.70 [1.36–5.38]**	**0.0184**	−36.47	−35.01	−34.28
		0	0	L	0.15	0.25	0.38 [0.20–0.71]	0.0302	−36.42	−34.97	−34.23
		0	2		0.02	0.08					
DOCK1*LAMA2	rs11017150*rs2244008	**0**	**0**	**L**	**0.38**	**0.71**	**0.21 [0.12–0.38]**	**4.12E-06**	−29.93	−5.401	−30.08
		1	0	H	0.30	0.14	4.93 [2.48–9.78]	1.98E-05	−25.92	−49.99	−26.06
		1	1		0.17	0.01					
PIP5K1C*FLNB	rs542690*rs1658338	1	1	H	0.34	0.18	2.72 [1.39–5.34]	0.0185	−37.67	−35.28	−36.19
		2	0	L	0.06	0.12	0.29 [0.13–0.63]	0.0333	−37.94	−35.55	−36.46
		1	2		0.03	0.08					

^a^ Category assigned to the genotype combination; H: increased RA risk and L: reduced RA risk.

^b^ *p*-value obtained from one million of permutations.

In bold: interactions replicated with categories identified in the initial sample.

## Discussion

Using whole-exome sequences from patients and healthy relatives belonging to multiplex families, we showed an accumulation of rare RA-associated variants in the genes participating in four biological functions: regulation of focal adhesions, the response to interferons, signaling through Rho GTPases, and lipid homeostasis. In addition, we report epistatic relationships between four genes whose products are involved in focal adhesion, the most significant pathway.

Although some uncertainty could be raised regarding the enrichment analysis results because of the nature of the variants considered (for example, intronic variant or SNV with a low prediction effect), the identification of functions previously established as participating in RA physiopathology supports the validity of the findings. Previous studies have described an increase in the type I interferon response in RA patients even at an early stage of the disease (Castañeda-Delgado et al., 2017; Muskardin and Niewold, 2018) and an association with response to therapy (Cooles et al., 2018; 2022; de Jong et al., 2019). In addition, Rho GTPase signaling is essential for several mechanisms involved in RA, from the cytokine production by immune cells to the dynamics of joint structural cells such as synoviocytes and osteoclasts (Nakayamada et al., 2005; Zeng et al., 2022). Recently, in a study analyzing the relationship between non-synonymous SNVs identified by WES in RA patients and disease activity, Chen and others identified key genes related to lipid metabolism through protein–protein interaction (PPI) results (Chen et al., 2023). In addition to the four biological functions, the enrichment analysis identified pathways associated with other disorders, including diseases associated with protein glycosylation and pathways involved in the pathogenesis of cardiovascular diseases (CVDs). Interestingly, CVD risk is substantially increased in RA (van Halm et al., 2009). Furthermore, to assess the causality of RA association with coronary artery disease (CAD), a subtype of CVD, a recent Mendelian randomization approach performed using European data showed that genetic liability to RA was associated with an increased risk of CAD (Qiu et al., 2021; Yuan et al., 2022). These results support the interest in studying shared risk factors between RA and CVDs.

The most significant result revealed the aggregation of RA rare variants in the pathway regulating the dynamics of focal adhesions. This pathway has already been identified from WES comparing 56 RA and 66 healthy controls in the Han Chinese population (Y. Li et al., 2017). These structures connecting the proteins from the extracellular matrix (ECM) to actin filaments of the cell cytoskeleton are involved in cell migration, differentiation, and survival. Their role in the pathogenesis of the disease has been supported by different studies that showed, for example, the requirement of focal adhesion kinase for synovial fibroblast invasion in RA patients (Shelef et al., 2014) and the role of vascular cell adhesion molecules in the interaction of synovial fibroblasts with immune cells (Postigo et al., 1992; Yoon and Moon, 2021). These observations and the results of our analysis lead us to further study this pathway and search for possible genetic interactions modulating the risk of developing RA. Note that this pathway has also been identified in other autoimmune diseases: in osteoarthritis and systemic lupus erythematosus from differentially expressed genes (Tang et al., 2012; He et al., 2016) and in type 1 diabetes from genes targeted by circulating miRNAs (Satake et al., 2018).

The MB-MDR approach was used to test the GxG interaction in the latter pathway since it has some advantages. It has been shown that MB-MDR has sufficient power to detect epistasis even if the data suffer from drawbacks such as missing data, genotyping error, genetic heterogeneity, population structure, or low sample size (Cattaert et al., 2011; Mahachie John et al., 2011; Abegaz et al., 2021). It also allows taking into account rare variants in the interaction test (Fouladi et al., 2015). Recently, this approach has been extended to empower individual risk prediction in personalized medicine. Simulation studies have demonstrated that this new algorithm outperforms random forest and elastic net approaches (Gola and König, 2021). We thus decided to use this method for our familial sample. Due to the small number of relatives in each family, relatedness was not considered in the analysis of the discovery set. Nevertheless, the replication set, consisting of unrelated cases and controls, leads to the validation of some interactions.

This statistical method allowed us to identify epistasis between two pairs of genes: MYLK*FLNB and DOCK1*LAMA2. The question of relation between *HLA-DRB1* shared epitope alleles and these interactions is arising in the context of RA patient carriers. When using different bioinformatics tools—HLA reporter (Huang et al., 2015) and SNP2HLA (Jia et al., 2013)—to investigate the *HLA-DRB1* status in controls, no conclusive results were obtained, essentially due to data quality (phasing issues and insufficient read depth). Consequently, the question could not be addressed (data not shown).

Concerning the SNVs involved in significant interactions, rs2244008, located in *LAMA2*, is a missense variant, whose effect is predicted to be low. Variants rs2605418 and rs1658338, respectively, located in *MYLK and FLNB*, are intronic. SNV rs11017150 is in a splice region of the *DOCK1* gene. In reviewing the functional impact of these four variants, data collected through the GTEx project highlighted significant quantitative trait loci (QTLs) for two SNVs out of four. However, correlated expression profiles are not suggestive of RA-specific tissue or functions. SNV rs2605418 is the only one of the four to be in a region of seven SNVs with strong linkage disequilibrium in the CEU population. The functional impact reported in the GTEx database for each SNV in this LD block is like rs2605418’s one (https://www.ebi.ac.uk/gwas/publications/36224396). Finally, none of them were associated with RA or a related phenotype through GWAS results. However, concerning *FLNB (filamin B)* gene, frequent variants have been associated with characteristics of bone structure in women (Wilson et al., 2009), which could be related to RA. From a functional point of view, the *LAMA2* gene encodes for the sub-unit α of laminins 2 and 4, which are proteins of the ECM mainly expressed in epithelial cells. The role of these proteins in RA physiopathology has not been defined yet. Nevertheless, *LAMA2* is hypo-methylated (Whitaker et al., 2013) and over-expressed (Sun et al., 2019; Zhang et al., 2019) in fibroblast-like synoviocytes of RA patients compared to patients affected by other arthritic or autoimmune diseases. Interestingly, the interaction between the LAMA2 protein and another protein of focal adhesions, namely, dedicator of cytokine 1 (*DOCK1*), promotes the migration of fibroblast-like synoviocytes, essential cells in RA, via *RAC1* and *ELMO1* gene’s product (Whitaker et al., 2015). This functional relation between *DOCK1* and *LAMA2* could be an illustration of the genetic interaction we observed.

To conclude, this interaction analysis allows identifying new genetic risk factors contributing to missing heritability in RA. In particular, we identified interactions implicating four genes of the focal adhesion pathways. Additional functional and omic evidence of the potential role of *FLNB*, *LAMA2*, and *DOCK1* exists to argue for further investigations.

## Data Availability

The original contributions presented in the study are publicly available. This data can be found here: https://www.ebi.ac.uk/eva/?eva-study=PRJEB57722.
