# Supplementary material for: MYLK*FLNB and DOCK1*LAMA2 gene–gene interactions associated with rheumatoid arthritis in the focal adhesion pathway
Source: Front Genet. 2024 May 13;15:1375036. doi: 10.3389/fgene.2024.1375036 (PMC11128622; doi:10.3389/fgene.2024.1375036)
Supplement: Supplementary file 1 [file Table1.DOCX]

**Table S1.** Biological pathways significantly enriched with genes including non-neutral rare variants with complete penetrance and no phenocopy in 9 multiplex families

| Pathway | FDR† | # tested genes | Genes |
| --- | --- | --- | --- |
| Focal adhesion | 0.0054 | 18 | *COL4A4, COL4A6, DIAPH1, FLNA, FLNC, FLT4, ITGB4, LAMA5, MET, PARVB, VCL, A2M, ADAMTS4, ADAMTS8, COL15A1, FBN2, CDH12, NECTIN3* |
| Antiviral mechanism by IFN-stimulated genes | 0.0081 | 6 | *EIF2AK2, EIF4A2, FLNA, NEDD4, NUP214, NUP98* |
| Hypothesised pathways in pathogenesis of cardiovascular disease | 0.0098 | 4 | *FBN2, FLNA, RUNX2, TGFBR2* |
| SREBF and miR33 in cholesterol and lipid homeostasis | 0.010 | 3 | *SIRT6, SREBF1, SREBF2* |
| Signaling by Rho GTPases | 0.0123 | 22 | *AURKB, CENPF, CENPL, CENPP, CKAP5, KIF2B, NUP98, SPDL1, A2M, ARHGAP22, ARHGAP25, ARHGEF33, CFTR, DIAPH1, DIAPH3, DLC1, FLNA, H2AFJ, NCKAP1L, CNTRL, TUBGCP5,* |
| Diseases associated with glycosylation of proteins | 0.0185 | 9 | *ADAMTS4, ADAMTS8, ADAMTSL4, ADAMTSL5, ALG8, LUM, THSD4, THSD7A, GALNT7* |

†: FDR calculated by applying Benjamini-Hochberg procedure
