# Supplementary material for: MYLK*FLNB and DOCK1*LAMA2 gene–gene interactions associated with rheumatoid arthritis in the focal adhesion pathway
Source: Front Genet. 2024 May 13;15:1375036. doi: 10.3389/fgene.2024.1375036 (PMC11128622; doi:10.3389/fgene.2024.1375036)
Supplement: Supplementary file 2 [file Table2.DOCX]

**Table S2.** GO pathways significantly enriched with genes including non-neutral rare variants with complete penetrance and no phenocopy in 9 multiplex families

| GO group | FDR† | # tested genes |
| --- | --- | --- |
| Cellular response to external stimuli | 0.0296 | 17 |
| Tissue morphogenesis | 0.0174 | 10 |
| Skeletal muscle myosin filament assembly | 0.0142 | 8 |
| Regulation of alpha-beta T cells | 0.0188 | 8 |
| Branching morphogenesis of an epithelial tube | 0.0171 | 7 |
| Regulation of cilium assembly | 0.0176 | 7 |
| Neural crest cell differentiation | 0.0140 | 6 |
| Lymphocyte homeostasis | 0.0143 | 6 |
| Regulation of ossification | 0.0397 | 6 |
| Vesicle transport along microtubule | 0.0178 | 5 |
| Autophagy of mitochondrion | 0.0365 | 5 |
| Microtubule bundle formation | 0.0388 | 5 |
| Cholesterol biosynthetic process | 0.0398 | 5 |
| Regulation of extrinsic apoptotic signaling pathway | 0.0237 | 4 |
| Regulation of tumor necrosis factor production | 0.0370 | 4 |
| Regulation of peptidyl-threonine phosphorylation | 0.0395 | 4 |
| Regulation of vascular associated smooth muscle cell migration | 0.0179 | 3 |
| Glucocorticoid receptor signaling pathway | 0.0258 | 3 |
| Sympathetic nervous system development | 0.0258 | 3 |
| Regulation of hormone biosynthetic process | 0.0361 | 3 |
| Chemokine receptor activity | 0.0393 | 3 |
| Regulation of stress fiber assembly | 0.0404 | 3 |

†: FDR calculated by applying Benjamini-Hochberg procedure
