# Supplementary material for: MYLK*FLNB and DOCK1*LAMA2 gene–gene interactions associated with rheumatoid arthritis in the focal adhesion pathway
Source: Front Genet. 2024 May 13;15:1375036. doi: 10.3389/fgene.2024.1375036 (PMC11128622; doi:10.3389/fgene.2024.1375036)
Supplement: Supplementary file 3 [file DataSheet1.pdf]

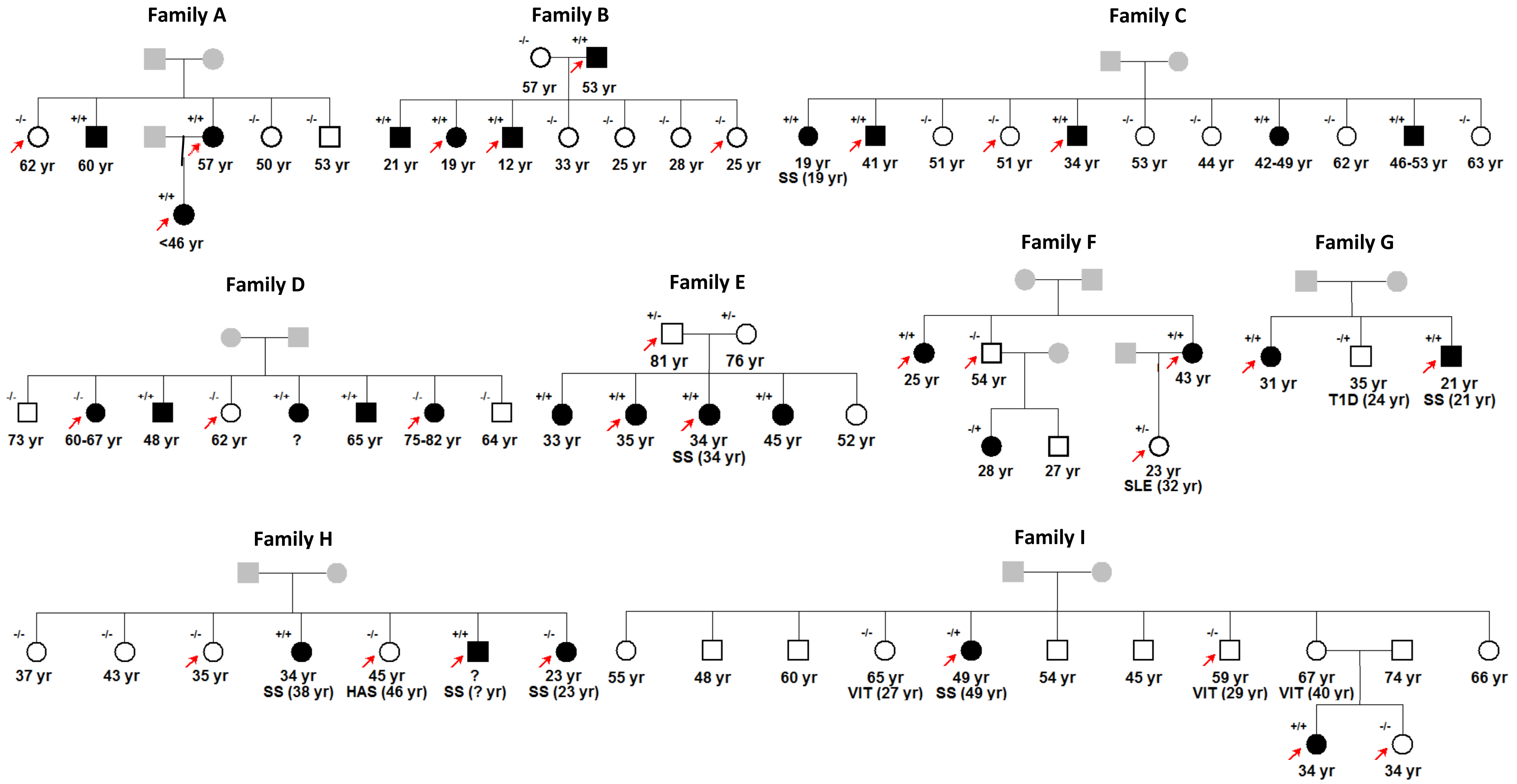

Figure S1. Pedigree charts of the 9 French families with multiple cases of autoimmune diseases included in the study.

Red arrows indicate family members sequenced by WES. Squares and circles denote males and females respectively. Color in symbols indicates the RA status: black for RA cases; blank for unaffected; and grey for unknown RA status. Text beside symbols indicates the ACPA and Rheumatoid factor (RF) status when it is known (+/+=positive for ACPA and RF; +/-=positive for ACPA and negative for RF; -/+=negative for ACPA and positive for RF; -/-=negative for ACPA and RF). First line below symbol s indicates the age at exam for unaffected and age at diagnosis for RA cases if known (?: unknown and a-b: age at diagnostic between a and b). Second line indicates other autoimmune disease (and age at diagnosis): SS = Sjögren's Syndrome; SLE = Systemic Lupus Erythematosus; T1D = TYPE 1 DIABETE; HAS = HASHIMOTO; VIT = Vitiligo.
