# Supplementary material for: MYLK*FLNB and DOCK1*LAMA2 gene–gene interactions associated with rheumatoid arthritis in the focal adhesion pathway
Source: Front Genet. 2024 May 13;15:1375036. doi: 10.3389/fgene.2024.1375036 (PMC11128622; doi:10.3389/fgene.2024.1375036)
Supplement: Supplementary file 4 [file Table3.DOCX]

**Table S3.** Assay's references for genotyped variants in the validation dataset.

| SNV | Assay ID (Thermo Fisher Scientific) |
| --- | --- |
| rs2605418 | C___1602695_10 |
| rs1658338 | C___8774085_10 |
| rs11017150 | C__25593452_10 |
| rs2244008 | C__16047108_10 |
| rs2229864 | C__16242465_10 |
| rs55956729 | C__25962638_10 |
| rs542690 | C___8353654_10 |
| rs2989519 | C___3022579_10 |
